# The longevity transporter mIndy (Slc13a5) as a target for treating hepatic steatosis and insulin resistance

**DOI:** 10.18632/aging.100907

**Published:** 2016-02-23

**Authors:** Diana M. Willmes, Stephen L. Helfand, Andreas L. Birkenfeld

**Affiliations:** ^1^ Section of Metabolic Vascular Medicine, Medical Clinic III, Dresden University School of Medicine, Technische Universität Dresden, Dresden, Germany; ^2^ Paul Langerhans Institute Dresden (PLID), German Center for Diabetes Research (DZD), Dresden, Germany; ^3^ Department of Molecular Biology, Cell Biology and Biochemistry, Brown University, Providence, RI 02912, USA

**Keywords:** INDY, Slc13a5, type 2 diabetes, fatty liver, aging, longevity

Reduced expression of the Indy (I'm Not Dead Yet) gene in Drosophila melanogaster and Caenhorabditis elegans extends lifespan in most [1-6], but not all [7], studies. Reducing Indy affects PGC-1α [4] [5] and AMPK signaling [6], leading to reduced whole body fat content and insulin-like proteins [2, 3, 6], as well as an increase in mitochondrial biogenesis [4]. Indy and its mammalian homolog mIndy (SLC13A5, NaCT) are transporters of TCA cycle intermediates, handling the uptake of citrate and other dicarboxylates via the plasma membrane into the cytosol [8, 9]. In mammals, deletion of mIndy protected mice from diet- and aging-induced obesity and insulin resistance.

The association of mIndy with fat and glucose metabolism in the liver has been further implicated by the demonstration that glucagon acts as a hormonal regulator of the mIndy gene, inducing mIndy expression via a CREB-dependent mechanism in rats [10]. Similarly, activation of the aryl hydrocarbon receptor (AhR) [11] induced mIndy in rat hepatocytes. Activation of AhR leads to fatty liver disease. Moreover, reducing mIndy in human hepatocytes with siRNA leads to a decrease in cellular lipid content. Together, these data suggested that hepatic mIndy is involved in the regulation of hepatic intermediary lipid and glucose metabolism and could be a useful target for the treatment of hepatic insulin resistance and fatty liver disease.

In a recent report in Aging, the targeting of mIndy as a therapeutic measure for reducing fatty liver has now been demonstrated in a mammalian model in vivo. Pesta et al. [12] investigated the effect of knockdown of mIndy using 2′-O-methoxyethyl chimeric anti-sense oligonucleotides (ASOs), an approach for reducing gene expression specifically in the liver [13]. The intervention reduced hepatic mIndy mRNA expression by 91%, and led to a significant reduction of liver fat content. Moreover, plasma triglycerides were reduced by 35% and overall and hepatic insulin sensitivity was improved, as assessed by the hyperinsulinemic-euglycemic clamp technique [12]. Interestingly, these benefits occurred independent of changes in body weight.

The approach chosen by Pesta et al. mimics a therapeutic setting, since the application of ASOs leads to an inducible knockdown of the target gene in vivo. It was not certain, however, that the reduction of citrate uptake by the deletion of mIndy was responsible for the beneficial effects on the liver. A new study by Huard et al. [14], describing the first competitive inhibitor of citrate uptake via mINDY suggests that the inhibition of carboxylic acid uptake is responsible for these beneficial metabolic effects. The novel compound described in this paper inhibited the uptake in a competitive and stereo-sensitive manner. At the same time, the approach offered complete protection from diet-induced glucose intolerance in mice and amelioration of diet-induced fatty liver disease after only 20 days of application [14]. These compounds were safe and well tolerated with no signs of neurological side effects. Taken together, the growing body of research nicely shows how findings can be successfully translated from D. melanogaster [1] to C. elegans [2, 6] to V. cholerae [9] to B. anynana [15], to mice [16], rats [12] pigs [17] and now human tissue [18]. The identification of a novel mINDY inhibiting compound is an important proof-of-concept demonstrating the therapeutic potential of mINDY inhibitors for the treatment of insulin resistance and non-alcoholic fatty liver disease. It will be of great interest to see whether or not these findings can be extended to a therapeutic setting in humans. The ultimate question then will be whether such a compound will also promote healthy aging and longevity.
